# Evaluation of the effect of magnetic field on rapeseed growth and the causal agent of blackleg disease, *Phoma lingam*

**DOI:** 10.5114/bta.2024.139754

**Published:** 2024-06-25

**Authors:** Samira Peighami Ashnaei, Reyhane Sadeghi, Laleh Hosseinian, Ahmad Shafaeizadeh, Mehran Zeinalipour, Hamid Keshvari, Mehrdad Imanzadeh, Mostafa Bahmanabadi

**Affiliations:** 1 Plant Disease Research Department, Iranian Research Institute of Plant Protection, Agricultural Research, Education and Extension Organization (AREEO), Tehran, Iran; 2 Health Technology Research Institute, Amirkabir University of Technology, Tehran, Iran

**Keywords:** *Leptosphaeria* species, magnetic fields, disease incidence, photosynthetic pigments

## Abstract

In recent years, with the increased production of oilseed rape, there has been a simultaneous enhancement in reports on pathogens causing diseases. Magnetic technology has been recognized as a new agricultural method aimed at improving health and crop production. In this work, the effect of magnetic fields was studied on the mycelial growth and conidia formation of *Leptosphaeria maculans* Gol125 and *Leptosphaeria biglobosa* KH36, the causal agents of Phoma stem cancer (blackleg) disease in rapeseed. In addition, seeds exposed to eight direct frequencies of magnetic fields were impregnated with pathogen suspension and grown under greenhouse conditions. The growth speed of both pathogen isolates decreased by 1–28% in GOL125 and 6–46% in KH36 over time in cultures exposed to magnetic fields. However, the number of conidia increased significantly under magnetic field exposure, reaching 5.4 × 10^7^ and 7.7 × 10^7^ SFU/ml in KH36 and GOL125 isolates, respectively. Furthermore, in greenhouse conditions, an increase in photosynthetic pigment levels was observed in almost all of the magnetic field-treated plants. In addition, disease incidence decreased by around 6% in the magnetic field-treated plants. This study represents the first evaluation of magnetic technology in controlling plant diseases. The use of magnetic fields may present a viable strategy for a sustainable production system; however, it requires further advanced studies to improve plant health and productivity.

## Introduction

Rapeseed (*Brassica napus* ) is the world’s second mostproduced oilseed, accounting for approximately 12% of the world’s major vegetable oil production (Zheng and Liu, 2021). In recent years, increasing the average yield of oilseed rape has significantly enhanced the profitability of this crop and expanded its cultivation area. However, a decline in the average yield of rapeseed has been observed in Canada, China, Europe, and Australia, coinciding with increasing occurrences of biotic stresses (Zhang et al., 2020). Oilseed rape is susceptible to many fungal pathogens, with *Phoma lingam* (teleomorph: *Leptosphaeria maculans*), the causal agent of blackleg disease, being one of major economic importance, causing millions of tons of rapeseed losses worldwide (Brachaczek et al., 2021). The introduction of synthetic fungicides in agrosystems changes the physiological, chemical, and microbiological balance in the soil (Fenta and Mekonnen, 2024). Therefore, developing sustainable agricultural systems may more effectively support our environment.

The environment is commonly exposed to different frequencies of electromagnetic fields (MFs), including TV towers, military and civil radiocommunication services, radar devices with frequencies above 100 MHz, and household appliances and power lines with extremely low frequencies (Pophof et al., 2023). Considering that short-term and long-term exposures may have significant effects on plants and related microbes, it is worth evaluating the impacts of magnetic technology in agriculture. Numerous studies have highlighted the importance of magnetic fields on various living organisms such as fungi and bacteria (Mohtasham et al., 2016; Masood et al., 2020); however, our knowledge about their effects on plants remains incomplete.

Only a few studies have demonstrated the impact of electromagnetic frequencies on agricultural microbiology in *in vitro* conditions. It has been shown that the number of developed conidia of *Alternaria alternata* and *Curvularia inaequalis* increased by 68–133%. However, at the same time, the number of *Fusarium oxysporum* conidia decreased by 79–83% after exposure to magnetic fields with 0.1, 0.5, and 1 MT flux densities (Nagy and Fischl, 2004). Ruzic et al. (1997) studied the effect of 0.025 and 0.1 mT magnetic flux densities on mycelial growth and ergosterol content of *Pisolithus tinctorius* fungus. They demonstrated that the stimulatory effect of a magnetic field of 0.1 mT flux density was quicker between the 7^th^ and 14^th^ days of treatment than that of a magnetic field of 0.025 mT. Also, the ergosterol content in the fungus slightly increased in the first week of treatments. A magnetic field of 200 mT flux density static and 29 mT flux density pulsating imposed morphological changes on the conidia of *Aspergillus puniceus* and *A. alternata*, as well as changes in colony pigmentation of *Aspergillus niger*. The fungus *A. niger* treated with a magnetic field of 10 mT showed a decrease in fungal cell conductivity from 1.78 × 10^-1^ to 0.51 × 10^-5^ Siemens/meter (Sadauskas et al., 1987). In fact, the changes in the ion signal channel of the microbial cell membrane included an increase in cell viability, and consequently, a change in cellular morphology (Lin et al., 2019). In addition, magnetic fields impacted the enzymatic activity of the microorganisms, either by promoting dehydrogenase activity or inhibiting tryptophan deaminase activity (Letuta et al., 2014). The activity of laccase in fungi has been improved by using a 10–40 Hz rotating MF (Zheng et al., 2013). The CMCase enzyme activity of *A. niger* decreased from 30 to 22.5 IU/ml after 2 h of exposure (Abo-Neima and El-Metwally, 2021). On the other hand, it was shown that static and sinusoidal 50 Hz MF (0.35 and 2.45 mT) did not induce alterations in the growth of the haploid yeast strain *Saccharomyces cerevisiae* WS8105-1C (Ruiz-Gomez et al., 2004). Cellini et al. (2008) studied the genetic effect of electromagnetic fields on bacterial DNA exposed to 50 Hz, and the results revealed no obvious differences among DNA patterns in each condition of study.

Some studies have examined the effects of magnetic fields on plant cells. MF treatment increased the chlorophyll content in sugar beet (*Beta vulgaris* L.) leaves (Hozayn et al., 2013) and the content of chlorophyll *a*, *b*, and carotenoids in potatoes (*Solanum tuberosum* L.) (Atak et al., 2007). Flórez et al. (2007) observed increased initial growth stages and early sprouting of maize and rice seeds exposed to the 125 and 250 mT stationary magnetic fields. Shabrangy et al. (2021) observed similar effects on the shoot and root proteome of barley plants under a magnetic field of 7 mT density. They showed that 38 proteins in the shoot and 15 proteins in the root changed significantly under the MF effect. For example, proteins involved in primary metabolic pathways increased, whereas proteins with a metal ion binding function decreased.

The aim of this work was to examine the effect of magnetic fields on the mycelial growth and conidia formation of *Leptosphaeria* species over a 10-day period. Furthermore, this is the inaugural study to reveal the potential effects of magnetic fields on plant–microbe interactions. We subjected rapeseed seeds exposed to the MF to determine whether magnetic technology stimulates or inhibits plant growth and the severity of disease caused by *P. lingam* (*L. maculans* ) under *in vivo* conditions.

## Materials and methods

### Isolation and identification of fungal isolates

Two *P. lingam* strains, Gol125 and KH36, were isolated from the collar and stem of rapeseed plants (winter rapeseeds showing blackleg symptoms) in the fields of southern Iran. The samples were sectioned and cultured on potato dextrose agar (PDA). Total DNA extraction followed the protocol published by Barnes et al. (2001). The polymerase chain reaction mixtures (PCR; 25 μl) contained 1 μl DNA, 1.25 μl of each primer (10 Mm), 17.5 μl of sterile deionized water, 0.5 μl of dNTPs (10 Mm), 0.2 μl of Taq DNA polymerase, 0.8 μl of MgCl_2_ (50 mM), and 2.5 μl of 10× buffer [200 mM Tris HCl (pH 8.4), 500 Mm KCl]. The internal transcribed spacer (ITS) region was amplified using the primers ITS5 (forward) 5′−(GGAAGTAAAAGTCGTAACAAGG) −3′ and ITS4 (reverse) 5′− (TCCTCCGCTTATTGATATGC) −3′, universal primers (White et al., 1990). The amplified sequences (600–700 bp) were compared with other sequences in the National Center for Biotechnological Information (NCBI) database using the Basic Local Alignment Search Tool (BLAST). The sequences of *L. maculans* Gol125 and *Leptosphaeria biglobosa* KH36 were deposited in GenBank (OK036810 and MZ948829, respectively).

### In vitro experiments

#### Fungal growth condition and magnetic field frequencies

The fungi were grown on PDA plates, and the inocula were taken from the growing zones of the cultures and then incubated at 25°C. Following the incubation of the cultures, those displaying uniform growth and morphology were selected and subjected to magnetic fields using an inductor and a ferrite ring core, all maintained at room temperature and humidity (25°C; *h*_r_ = 50%) for both the control and treated cultures. A voltage of 20 volts was applied to both ends of the coil, with a current of about 20 mA passing through it. The direct electromagnetic frequencies were applied in two different sets: 481, 544, 3966 Hz (denoted as factor A) and 30, 31, 29 KHz (denoted as factor B), each for 12 h (a day), over a total period of 60 min with 3-min intervals. The total induced energy was approximately 4 J (joules).

### Data recorded

#### Mycelial growth and sporulation of the fungi

The diameters of mycelial growth in each culture were measured every 24 h in two perpendicular directions for 10 days (Nagy and Fischl, 2004). The average of these two diameters was considered as the culture’s diameter, and this average was calculated based on three repetitions. The developed conidia of *L. maculans* Gol125 and *L. biglobosa* KH36 were counted using a hemocytometer per milliliter of suspension after 10 days of growth. To do this, 10 ml of sterilized water was added to the Petri dishes, and the conidia were counted by adding 10 μl of conidium suspension to each side (16 subcells of 0.062 mm^2^) of the hemocytometer. The experiments were repeated three times.

### In vivo experiments

#### Plant growth conditions and magnetic field frequencies

Rapeseed plants of the Westar cultivar (prepared from the Iranian Research Institute of Plant Protection) were grown in 1 kg pots containing a mixture of sand, pit, and perlite (in a ratio of 1:70:29) at 21/16°C and 95% humidity, within a greenhouse environment (five plants per pot with three replicates). Before sowing, the seeds underwent exposure to eight direct frequencies, including 29, 30, and 31 KHz, 12/5, 36, 42/75, 59/80, and 62 Hz, for 59 min each frequency (maintaining room temperature and humidity at 25°C; *h*_r_ = 50%, respectively), and were instantly planted thereafter.

#### Pathogen growth conditions and pathogen infection

The culture of *L. maculans* Gol125 was grown on PDA plates and then incubated at 23°C for 72 h. The seeds exposed to MFs and the controls (not exposed to MFs) were impregnated with the pycnidiospore suspension (2 × 10^7^ spores/ml using a hemacytometer) of Glo125 before planting (Keypoor et al., 2015). The mock treatment involved seeds inoculated with sterilized distilled water. Therefore, four treatments were evaluated in the greenhouse assay: MF seeds and control seeds, both with the pathogen and mock seeds without the pathogen.

### Data recorded

#### Growth parameters, water capacity, and photosynthetic pigments content in rapeseed

At the six-leaf stage of rapeseeds, three plants from each treatment were evaluated based on shoot and root dry weight. Water content was determined according to Henson et al. (1981) using the following formula:

WC = 100 (fresh mass − dry mass)/fresh mass.

The chlorophyll *a*, chlorophyll *b*, and carotenoid contents in fresh leaves were estimated using the method outlined by Lichtenthaler and Buschmann (2001) with three replicates. Fresh tissue (50 g) from plants in each treatment was finely ground using mortar and pestles with 15 ml of 80% acetone. The optical density (OD) of the obtained solution was recorded at 662 nm (for chlorophyll *a*), 645 nm (for chlorophyll *b*), and 470 nm (for carotenoids) using a spectrophotometer (Shimadzu UV-1700, Tokyo, Japan). The values of photosynthetic pigments were expressed in mg/100 g FW (Hozayn et al., 2016).

#### Disease incidence

Disease incidence (DI) was measured by counting infected plants with round to irregular creamish-grey lesions on the leaves (at the 4–6 leaf stage) and calculating their percentage relative to the total number of plants in each treatment (Sosnowski, 2002). The experiment was replicated three times.

### Statistical analysis

A comparison between treatments, each with three biological replicates, was performed by analysis of the least-significant-difference (LSD) test (Webster, 2007) with SAS software. Mean differences were considered statistically significant at the 5% level.

## Results

### Mycelial growth of fungi in vitro condition

**Fig. 1:**
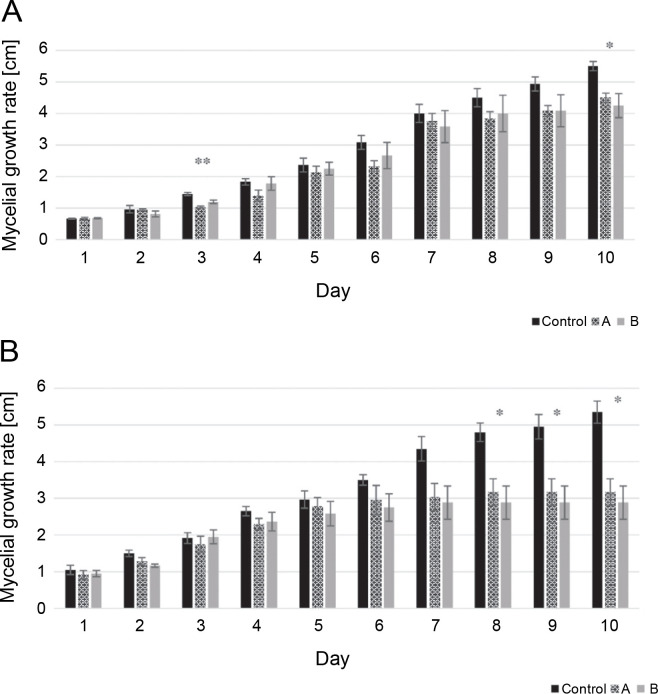
Mycelial growth rate (cm) in two isolates of *Phoma* spp. over 10 days; A) mycelial growth rate in *Leptosphaeria maculans* GOL125, B) mycelial growth rate in *Leptosphaeria biglobosa* KH36; A: electromagnetic frequency of 81, 544, 3966 Hz, B: electromagnetic frequency of 30, 31, 29 KHz; bars represent LSD for comparison between treatments with three replicates (*P < 0.05, **P < 0.005)

The mycelial growth rate of two isolates, including *L. maculans* Gol125 and *L. biglobosa* KH36, under MF conditions, is presented in Figure 1 alongside the control. The data showed that the growth rate of both isolates decreased over time in cultures exposed to magnetic fields compared to the control. Significant decreases were noted on the 8^th^, 9^th^, and 10^th^ days in *L. biglobosa* KH36 (Fig. 1B). The most significant decreases in KH36 isolate exposed to A and B MFs were observed, averaging 40 and 46%, respectively, on the 10^th^ day compared to the control (Table 1B). Likewise, significant decreases in the mycelial growth of *L. maculans* Gol125 exposed to magnetic fields were observed on the 3^rd^ and 10^th^ days compared to the control (Fig. 1A). The relative decreases in the mycelial growth of Gol125 exposed to A and B MFs were an average of 28 and 17% on the 3^rd^ day, and 18 and 22% on the 10^th^ day (Table 1A), respectively, compared to the control.

**Table 1:** Average reduction in mycelial growth: A – *Leptosphaeria maculans* Gol125, and B – *Leptosphaeria biglobosa* KH36, in percent (%), compared to the control

A – Leptosphaeria maculans GOL125			B – Leptosphaeria biglobosa KH36		
Day	A	B	Day	A	B
_1_^st^	–	–	_1_^st^	12.6	9.5
_2_^nd^	1.7	15.5	_2_^nd^	14.4	22.2
3^rd^**	28.7	17.26	_3_^rd^	8.3	–
_4_^th^	23.6	2.7	_4_^th^	13.2	10.6
_5_^th^	9.8	4.9	_5_^th^	6.1	12.9
_6_^th^	24.3	13.5	_6_^th^	15.7	21.4
_7_^th^	6.2	10.4	_7_^th^	30.2	33.7
_8_^th^	14.8	11.1	_8_^th^_*_	34	39.9
_9_^th^	17.2	17.2	_9_^th^_*_	36	41.7
10^th^*	18.1	22.72	10^th^*	40.8	46.1

A – magnetic fields of 81, 544, 3966 Hz, B – magnetic fields of 30, 31, 29 KHz (*P < 0.05, **P < 0.005); data were calculated based on three replicates

### Fungal sporulation

**Fig. 2:**
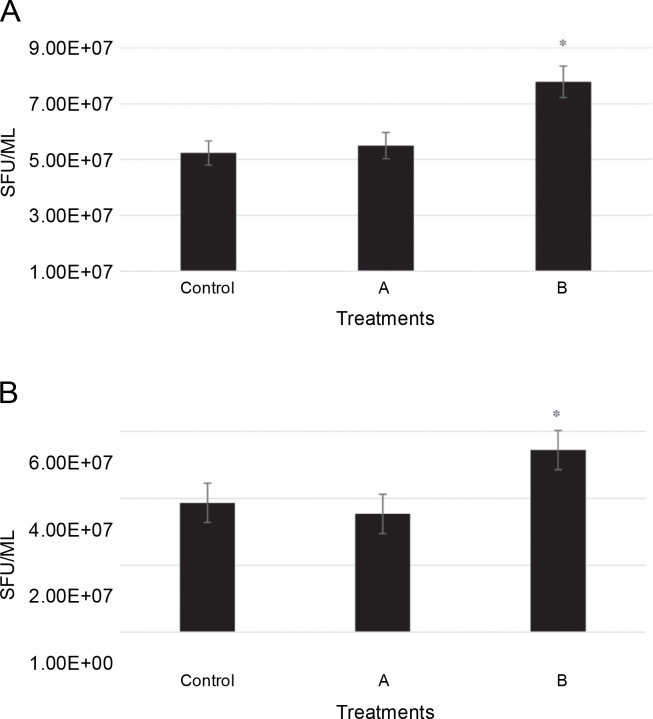
Number of conidia (SFU – Spore Forming Unit/ml) in two isolates of *Phoma* spp. on PDA medium after 10 days; A) the number of conidia (SFU/ml) in *Leptosphaeria maculans* Gol125, B) the number of conidia (SFU/ml) in *Leptosphaeria biglobosa* KH36; A: electromagnetic frequency of 81, 544, 3966 Hz, B: electromagnetic frequency of 30, 31, 29 KHz; bars represent LSD for comparison between treatments with three replicates, *P < 0.05

The number of conidia for two isolates, including *L. maculans* Gol125 and *L. biglobosa* KH36, under MF conditions, is illustrated in Figure 2 alongside the control. Conidia formation in *L. maculans* Gol125 increased after exposure to both A and B MFs compared to the control, with a significant difference observed in cultures exposed to B MF (Fig. 2A). The average number of conidia in the Gol125 isolate in B and control treatments was approximately 8 × 10^7^ and 5 × 10^7^ Spore Forming Units (SFU)/ml, respectively (Fig. 2A). A and B MFs impacted the conidia formation of *L. biglobosa* KH36 differently. The number of conidia in the KH36 isolate increased significantly in cultures exposed to B MF, while it decreased in cultures treated with A MF (Fig. 2B). The average number of conidia in B and control treatments was approximately 5.5 × 10^7^ and 4 × 10^7^ SFU/ml, respectively (Fig. 2B).

### Growth parameters and water capacity of rapeseed plants in vivo conditions

**Fig. 3:**
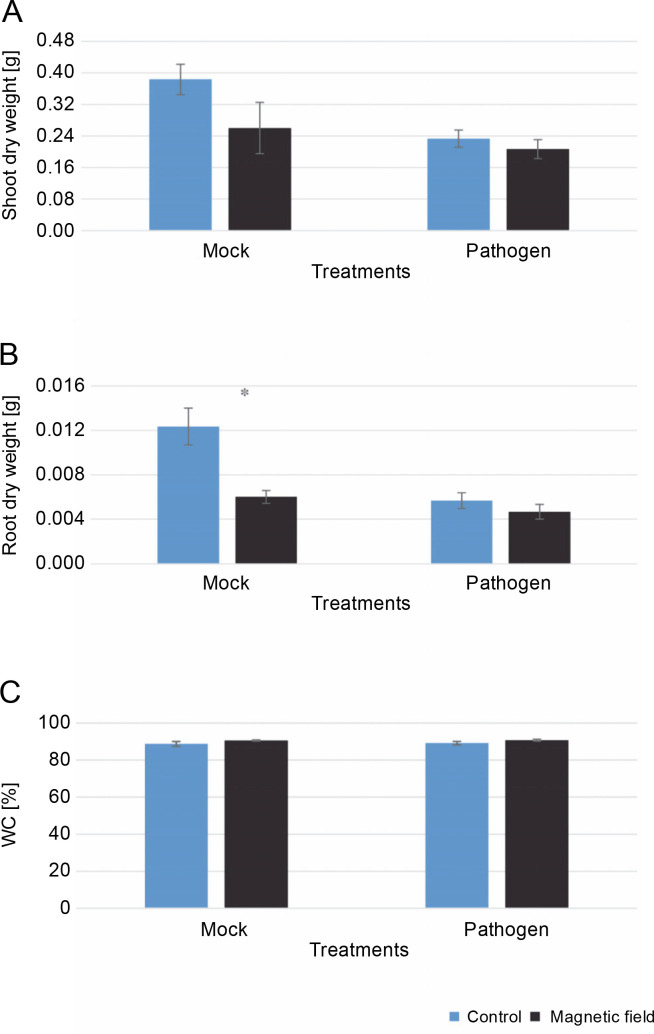
Effect of magnetic fields on rapeseed plants (Westar cultivar) grown in greenhouse conditions; A) shoot dry weight, B) root dry weight, C) the percentage of water capacity; Mock – control and magnetic field treatments without the pathogen (*Leptosphaeria maculans* Gol125), Pathogen – control and magnetic field treatments inoculated with a suspension of 2 × 10^7^ spores/ml of Gol125; magnetic field seeds were exposed to eight direct frequencies including 29, 30, 31 KHz, 12/5, 36, 42/75, 59/80, and 62 Hz; bars represent LSD (*P* < 0.05) for comparisons between treatments with three replicates

The effects of MFs and *L. maculans* Gol125 on the growth (root and shoot dry weight) and water capacity of rapeseed plants are shown in Figure 3. Surprisingly, in the greenhouse assay, a decrease in the shoot and root dry weights was observed in plants treated with magnetic fields compared to the control (Fig. 3A, Fig. 3B). This reduction was more dramatic and significant in the root dry weight of MFs plants without the pathogen (mock) compared to the control (Fig. 3B). The averages of root dry weights in mock treatments were 0.006 and 0.01 g in the magnetic field and control plants, respectively (Fig. 3B). Furthermore, there was a 2% increase in water capacity in MFs plants (both with and without *L. maculans* Gol125) compared to the control (Fig. 3C). The water capacity values in the MFs and control plants were evaluated at 91 and 89%, respectively, without any significant difference (Fig. 3C).

### Photosynthetic pigments of rapeseed plants in vivo conditions

**Fig. 4:**
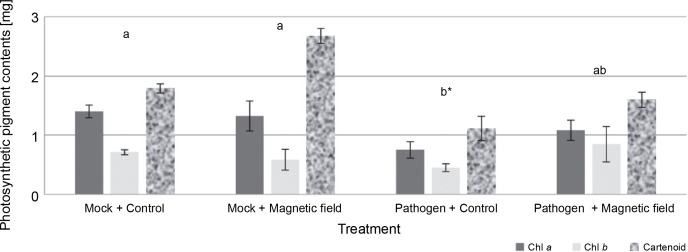
Photosynthetic pigments (including chlorophyll *a*, chlorophyll *b*, and carotenoids) in rapeseed plants, Westar cultivar; Mock – control and magnetic field treatments without the pathogen (*Leptosphaeria maculans* Gol125), Pathogen – control and magnetic field treatments inoculated with a suspension of 2 × 10^7^ spores/ml of Gol125; magnetic field seeds were exposed to eight direct frequencies including 29, 30, 31 KHz, 12/5, 36, 42/75, 59/80, and 62 Hz; bars represent LSD (*P* < 0.05) for comparisons between treatments with three replicates

The effects of MFs and *L. maculans* Gol125 on the photosynthetic pigments (chlorophyll *a*, chlorophyll *b*, and carotenoids) in rapeseed plants are presented in Figure 4. An increase in photosynthetic pigment levels, including carotenoids, chlorophyll *a*, and *b*, was observed in almost all of the magnetic field-treated plants (Fig. 4). The level of carotenoid pigments in MFs plants (with or without the pathogen) enhanced considerably compared to the control plants. Carotenoid levels were approximately 1.80 and 2.70 mg/50 g fresh weight in the mock treatments, and in the presence of *L. maculans* Gol125, about 1.1 and 1.60 mg/50 g fresh weight in MFs and control plants, respectively (Fig. 4). The lowest levels of photosynthetic pigments, including chlorophyll *a*, chlorophyll *b*, and carotenoid (1.08, 0.84, and 1.59 mg/50 g, respectively) fresh weight, were observed in the control plants infected with the pathogen (Fig. 4). Photosynthetic parameters are considered good criteria for monitoring the changes induced by stress.

### Disease incidence

**Fig. 5:**
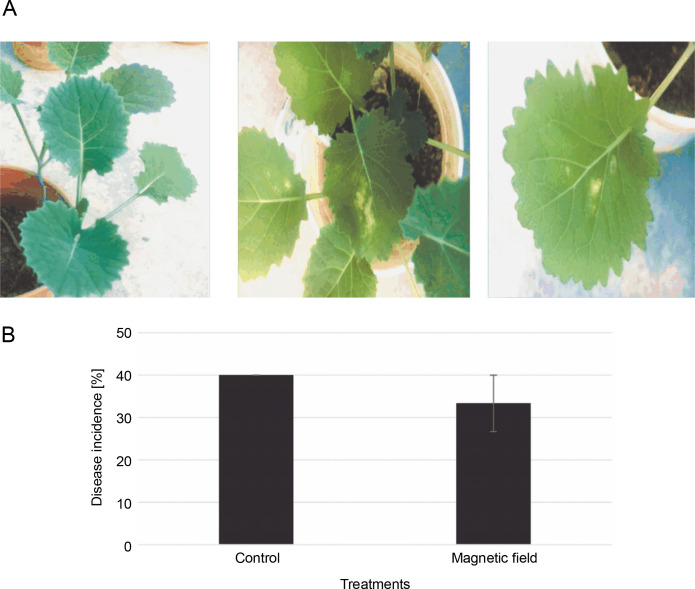
A) Disease symptoms caused by *Leptosphaeria maculans* Gol125 in rapeseed plants at the stage of 4/6 leaves – from left to right: control and plants infected with Gol125; B) disease severity in rapeseed plants inoculated with Gol125; magnetic field and control seeds were inoculated with a suspension of 2 × 10^7^ spores/ml of Gol125; magnetic field seeds were exposed to eight direct frequencies including 29, 30, 31 KHz, 12/5, 36, 42/75, 59/80, and 62 Hz; bars represent LSD (*P* < 0.05) for comparisons between treatments with three replicates

The symptoms of the disease (irregular creamish lesions on the leaves) were detectable at the 4–6 leaf stage (Fig. 5A). DI decreased by around 6% in MFtreated plants; however, there was no significant difference compared to the control (Fig. 5B). Disease severities were scored at about 34 and 40% in the MF-treated and control plants, respectively (Fig. 5B).

## Discussion

In this study, the mycelial growth rate of *Phoma* isolates exposed to magnetic fields decreased over time in *in vitro* conditions. Nagy and Fischl (2004) showed that the application of MFs decreased the growth of colonies of some pathogenic fungi, including *A. alternata*, *C. inaequalis*, and *F. oxysporum*, by 10%. However, the results obtained in this study contradict findings from some previous literature data. For instance, the average growth of the mycorrhizal fungus *P. tinctorius* exposed to magnetic fields was approximately 13% greater than that of the control (Ruzic et al., 1997). The use of lowfrequency MFs increased the mycelial growth of *Trichoderma asperrelloides* and *Pisolitbus microcarpus* (an ectomycorrhizal fungi) (Steffen et al., 2020). Interestingly, increases in mycelial growth were observed in beneficial microorganisms, highlighting the importance of microbe type in this context. The mycelial biomass and triterpenoid production in the fungus *Ganoderma lucidum* increased by 26.99% and 33.62%, respectively (Sun et al., 2021). The mycelial biomass of *Phellinus igniarius* improved by 22.64% using low-intensity ultrasound (Zhang et al., 2014). It was indicated that MFs can impact microbial metabolism processes through changes in cell surface hydrophilicity (a parameter that controls cell behavior via protein absorption) and alterations in cell membrane potentials (Bhargavi et al., 2018; Flimban et al., 2019). Therefore, it is important to meticulously control parameters such as MF intensity, exposure protocol, exposure time, and even time intervals before and after exposures to achieve the desired results.

In addition, the formation of conidia in both GOL125 and KH36 isolates increased under the B frequencies of MFs *in vitro* conditions. Considering the different effects of magnetic fields on fungi, Nagy and Fischl (2004) showed that the number of conidia in *A. alternata* and *C. inaequalis* increased by 68–133%, while the number of conidia in *F. oxysporum* decreased by 79–83%. The various impacts of magnetic fields on microbial morphology and activity were reviewed by Huang et al. (2017). It was shown that the simultaneous use of ultrasound and heat doubled the spore thermal inactivation rate in *Clostridium perfringens* bacteria (Evelyn and Silva, 2015). Despite the significance of microorganisms in agriculture, the number of publications covering magnetic fields in fungi (both beneficial and detrimental) is small compared to similar reports on humans.

In this study, growth parameters, water capacity, photosynthetic pigments, and DI in rapeseed plants were evaluated under MFs exposure and in the presence of the pathogen GOL125 in greenhouse conditions. A decrease in shoot and root dry weights, alongside an increase in photosynthetic pigment levels, was observed in plants treated with magnetic fields. It has been shown that root meristem proliferation was reduced, and RNA and protein synthesis were suppressed in *Pisum sativum*, *Linum usitatissimum*, and *Lens culinaris* under conditions of geomagnetic field shielding (Fomichjova et al., 1992). Inhibition of root growth was observed in *Glycine soja*, *Triticum aestivum*, and *L. culinaris* exposed to a magnetic flux density of 17.6 mT (Penuelens et al., 2004). Morillo-Coronado et al. (2022) showed that low-intensity stationary magnetic fields did not significantly affect seed germination, dry weight, or fresh weight of *Allium cepa* plants. However, the results obtained here contradict some other studies. For instance, MFs ranging from 0.06 to 0.36 T improved the early growth stages in *L. culinaris* plants, and the levels of stress enzymes including superoxide dismutase (SOD) and ascorbate peroxidase (APX) increased in the seedlings (Shabrangy and Majd, 2009). Early growth of maize and soybean plants was enhanced by using 200 mT MFs for 1 h, which also increased water uptake in these plants (Kataria et al., 2017). A magnetic field of 1–2 mT improved seedling growth, as well as fresh and dry weights of *Valeriana officinalis* plants (Farzpourmachiani et al., 2013). In seedlings of *Linum bienne*, root and shoot growth were stimulated by magnetic fields of 0–2 mT and 200–350 mT, while inhibited at 100–170 mT relative to the control (Belova and Lednev, 2001). It has been shown that very low magnetic fields can induce ultrastructural changes in meristematic root cells of *P. sativum* plants, such as the accumulation of lipid bodies and size increase of mitochondria (Belyavskaya, 2001), which may affect water absorption and subsequently water capacity. However, some previous studies showed a considerable effect of magnetic technology on water capacity in different plants. For example, water efficiency in canola plants increased significantly by irrigation with magnetic water, by 19.05% compared to the control plants (Hozayn et al., 2016). Selim and El-Nady (2011) indicated that magnetically treated tomato seeds, irrigated with magnetized water, could significantly overcome the adverse effects of water deficit. Generally, the contradictory outcomes of the studies indicate that the effects of MFs on plants may depend on plant species and MF characteristics such as intensity and exposure time (Nyakane et al., 2019). Therefore, choosing a precise frequency of the magnetic field based on the type of plant is important for the purposeful application of this technology.

Regarding photosynthetic pigments, the results obtained here confirm the observations of a previous study by Hozayn et al. (2016), where the percentage increments reached 8.86%, 22.22%, and 19.71% in chlorophyll *a*, chlorophyll *b*, and carotenoids, respectively, using magnetic water. Increases in chlorophyll contents have been reported in different plants exposed to MFs, such as potatoes and sugar beet (Atak et al., 2003; Hozayn et al., 2013); however, the exact mechanism is often unclear. Previously, it was shown that electromagnetic frequencies can affect cellular function through the stimulation of ion channels (Sahebjami et al., 2007; Lin et al., 2019). The stimulating effects of MFs on photosynthetic pigments of plants may be due to enhancing proline content, which increases some ions like Mg^+2^ needed for chlorophyll synthesis or K^+^ by increasing the number of chloroplasts per cell (Shaddad, 1990; Garcia-Reina and Arza, 2001). The increase in the levels of photosynthetic pigments can be related to the increase in cytokinin production due to the magnetic fields (Atak et al., 2003), since cytokinins play an important role in chloroplast development. Therefore, deciphering the related mechanisms may help achieve a precise frequency magnetic field in different plants.

To the best of our knowledge, this is the first study evaluating magnetic technology in plant-pathogen interactions in the greenhouse. Certain critical factors, such as soil conditions, plant types, and microbial populations, should be taken into account when studying the application of magnetic technology in the field. Since plants in natural conditions interact with biotic and abiotic factors that play critical roles in plant health and productivity (Fischer and Peighami Ashnaei, 2019), magnetic technology should be applied more precisely in field settings.

## Conclusion

The magnetic field has become an integral part of our environment and a source of energy. Directing it toward the desired aims, such as improving crop quality and quantity and managing biotic and abiotic stresses, may be useful for reaching sustainable agriculture. Given that most technologies used in agriculture depend on synthetic molecules to control phytopathogenic microorganisms, the use of innovative methods in this area may represent an environmentally friendly system to save the crops. In this study, different densities of MFs applied to two isolates of fungi reduced mycelial growth within a specified time frame. In addition, MF-treated seeds exhibited an increase in photosynthetic pigments, although similar results were not observed in root/shoot dry weights. Therefore, determining the specific and effective frequencies of magnetic fields, along with developing a precise protocol based on plant species and the type of microorganism, are fundamental and important steps that still require further advanced studies.
